# Clinical and virological factors associated with gastrointestinal symptoms in patients with acute respiratory infection: a two-year prospective study in general practice medicine

**DOI:** 10.1186/s12879-017-2823-9

**Published:** 2017-11-22

**Authors:** Laetitia Minodier, Shirley Masse, Lisandru Capai, Thierry Blanchon, Pierre-Emmanuel Ceccaldi, Sylvie van der Werf, Thomas Hanslik, Remi Charrel, Alessandra Falchi

**Affiliations:** 1 EA7310, Laboratoire de Virologie, Université de Corse-Inserm, 20250 Corte, France; 2 Institut Pierre Louis d’Epidémiologie et de Santé Publique, Sorbonne Universités, UPMC Univ Paris 06, UMR_S 1136, 56, Boulevard Vincent Auriol, 81393-75646 Paris, France; 3 INSERM, UMR_S 1136, Institut Pierre Louis d’Epidémiologie et de Santé Publique, 56, Boulevard Vincent Auriol, 81393-75646 Paris, France; 4 Pasteur Institute, Virology Department, Epidemiology and Physiopathology of Oncogenic Viruses Unit, F-75015 Paris, France; 5 UMR CNRS 3569, 75015 Paris, France; 6 Sorbonne Paris Cité, Cellule Pasteur, Université Paris Diderot, Institut Pasteur, 75015 Paris, France; 7 Pasteur Institute, Virology Department, Molecular Genetics of RNA Viruses Unit, F-75015 Paris, France; 8 Université Paris Diderot, Sorbonne Paris Cité, Unité de Génétique Moléculaire des Virus à ARN, EA302, F-75015 Paris, France; 9 Sorbonne Université, UPMC Université Paris 06, Institut Pierre-Louis d’Épidémiologie et de Santé Publique (IPLESP UMRS 1136), Paris, France; 10 Hôpital Ambroise Paré, service de médecine interne, Boulogne-Billancourt, France; 11 UFR des Sciences de la Santé Simone-Veil, Université Versailles Saint Quentin en Yvelines, Versailles, France; 12 UMR “Emergence des Pathologies Virales” (EPV: Aix-Marseille Univ - IRD 190 - Inserm 1207 - EHESP) & Fondation IHU Méditerranée Infection, APHM Public Hospitals of Marseille, Marseille, France

**Keywords:** Acute respiratory infection, Gastrointestinal symptoms, Enteric pathogens, Influenza virus, General practitioner

## Abstract

**Background:**

Gastrointestinal (GI) symptoms, such as diarrhea, vomiting, abdominal pain and nausea are not an uncommon manifestation of an acute respiratory infection (ARI).

We therefore evaluated clinical and microbiological factors associated with the presence of GI symptoms in patients consulting a general practitioner (GP) for ARI.

**Methods:**

Nasopharyngeal swabs, stool specimens and clinical data from patients presenting to GPs with an ARI were prospectively collected during two winter seasons (2014-2016). Samples were tested by quantitative real-time PCR for 12 respiratory pathogen groups and for 12 enteric pathogens.

**Results:**

Two hundred and four of 331 included patients (61.6%) were positive for at least one respiratory pathogen. Sixty-nine stools (20.8%) were positive for at least one pathogen (respiratory and/or enteric). GI symptoms were more likely declared in case of laboratory confirmed-enteric infection (adjusted odds ratio (aOR) = 3.2; 95% confidence interval [CI] [1.2–9.9]; *p* = 0.02) or human coronavirus (HCoV) infection (aOR = 2.7; [1.2–6.8]; *p* = 0.02). Consumption of antipyretic medication before the consultation seemed to reduce the risk of developing GI symptoms for patients with laboratory-confirmed influenza (aOR = 0.3; [0.1–0.6]; *p* = 0.002).

**Conclusions:**

The presence of GI symptoms in ARI patients could not be explained by the detection of respiratory pathogens in stools. However, the detection of enteric pathogens in stool samples could explained by the presence of GI symptoms in some of ARI cases. The biological mechanisms explaining the association between the presence of HCoVs in nasopharynx and GI symptoms need to be explored.

**Electronic supplementary material:**

The online version of this article (10.1186/s12879-017-2823-9) contains supplementary material, which is available to authorized users.

## Background

Gastrointestinal (GI) symptoms, such as diarrhea, vomiting, abdominal pain and nausea are not an uncommon manifestation of an acute respiratory infection (ARI) [1–8] (Additional file 1) and have been reported as a hallmark of severe influenza in childhood [9].

Influenza viruses and other respiratory agents such as human rhinoviruses (HRV) [10], have been detected in stools of patients with ARIs (Additional file 2) [5, 10–14], but their correlation with GI symptoms and their viability in stool is still debated [10, 15].

There are several possible explanations for the observed GI symptoms during an ARI. First, each winter, ARIs and gastroenteritis outbreaks overlap, creating a spurious association between ARI and GI symptoms, maybe caused by a co-infection between respiratory agents and enteroviruses [16]. Second, GI symptoms may be a side effect of drug treatment (antibiotic or antiviral) [17, 18] or food consumption (*ex: raw shellfish and molluscs*) [19]. Third, GI symptoms could either be a manifestation of a direct viral effect, or an indirect viral effect of respiratory viruses, such as lung-derived CD4+ cell-induced dysbiosis resulting in intestinal injury [20].

Insufficient information about the prevalence of GI symptoms in ARIs, their clinical features and their potential risk factors may lead to diagnostic errors and inadequate treatment.

In the context of the above limitations, the main objectives of this two-year (2014–2016) prospective study were to evaluate the demographical, clinical and microbiological factors associated with the presence of GI symptoms in patients presenting to general practitioner (GP) with an ARI.

## Methods

### Study design

A representative sample of 60 GPs from the French *Sentinelles* Network [21, 22] was recruited to enrol ARI patients from all over mainland France.

To ensure that the selection of ARI patients remained random, each GP was required to include, each week, the first two patients seen in consultation who met the inclusion criteria, regardless of their age. The case definition of ARI was “any person with a sudden onset of symptoms and at least one of the following four systemic symptoms: fever (≥ 38 °C or greater) or history of fever (≥ 38 °C or greater) taken at home or feverishness, malaise, headache, myalgia, AND at least one of the following three respiratory symptoms: cough, sore throat, or shortness of breath” [23]. All patients were recruited within 48 h of the onset of symptoms.

Patients were enrolled by their GPs over two consecutive ARI seasons from week 46, 2014 (10–16 November 2014) to week 15, 2015 (06-12 April 2015) and from week 45, 2015 (02-8 November 2015) to 16, 2016 (18–24 April 2016) (Additional file 3).

The GPs completed a case report form (CRF) for all volunteers who met the case definition and agreed to participate, and submitted this by post (all items are listed on Additional file 4). We defined a patient as vaccinated if he/she had received at least one dose of seasonal influenza vaccine at least 15 days before the onset of ARI symptoms.

### Sample collection

Two types of samples were obtained for each enrolled patient: a nasopharyngeal swab and a stool sample. The nasopharyngeal specimen was collected by the GP and was sent with the CRF to the test laboratory by post in a triple-packaged Copan universal transport medium (UTM-RT) container (Copan Italia, Brescia, Italy). Included patients were asked to collect stool specimens and send them to the laboratory within 48 h by post in triple packaging as required by the United Nations class 6.2 specifications.

### Laboratory investigations

#### Nucleic acid extraction

For nasopharyngeal specimens, nucleic acids were extracted from 200 μl of UTM-stored sample and eluted in 60 μl of elution buffer using QiAamp MinElute virus spin kits (Qiagen, France) according to the manufacturer’s instructions. Stool specimens were centrifuged at 14,000 xg for 20 min; then nucleic acids were extracted from 200 μl of the UTM-stored sample and eluted in 40 μl of elution buffer using QiAamp MinElute virus spin kits (Qiagen) according to the manufacturer’s instructions. An internal control (T4 and MS2 phages) was added to each extraction tube to assess the quality of the extraction at the end of the amplification [24].

### Detection of respiratory pathogens

All extracted samples (nasopharyngeal and stool) were screened for influenza A and B viruses by real*-*time quantitative Reverse Transcription PCR (RT-qPCR); influenza A virus-positive specimens were subtyped and influenza B virus-positive samples were analysed for Victoria and Yamagata lineage according to the method developed by the French National Influenza Centre [25, 26]. Then, the presence of 10 non-influenza respiratory pathogen groups was analysed by RT-qPCR and qPCR using a Fast Track Diagnostic (FTD) Respiratory pathogens 21 kit (Fast Track Diagnostic, Luxemburg) using five tubes containing primer and probe mix for different viruses; Tube-1 [*Influenza A*, *Influenza A* subtype H1N1 (A(H1N1)pdm09), human *Rhinovirus* (HRV), *Influenza B*], Tube-2 [human *Coronaviruses* NL63 (HCoV-NL63), 229E (HCoV 229E), OC43 (HCoV-OC43), and HKU1 (HCoV HKU1)], Tube-3 [human *Parainfluenza* viruses, 2, 3, and 4 (HPIV- 2, 3 and 4) & Internal Control], Tube-4 [human *Parainfluenza* viruses-1, Mycoplasma *pneumoniae* (M.pneu), human *Bocavirus* (HBoV), human *Metapneumovirus* (HMPV A/B)] and Tube-5 [*Respiratory Syncytial virus* (RSVA/B), human *Adenovirus* (HAdV), *Enterovirus* (EV), human *Parechovirus* (HPeV)].

### Detection of enteric pathogens

Extracted stool samples were screened by RT-qPCR and qPCR using the FTD Viral gastroenteritis kit (Fast Track Diagnostic, Luxemburg) according to the manufacturer’s instructions, using three multiplex PCRs to detect six viruses: human norovirus (hNoVG1 and hNoVG2), adenovirus (hAdV), human astrovirus (HAstV), rotavirus (RV) and sapovirus (SaV). The panel FTD Bacterial gastroenteritis kit (Fast Track Diagnostic, Luxemburg) was used following the manufacturer’s procedure, using two multiplex RT-qPCR for six bacteria: *Campylobacter coli/jejuni/lari, Escherichia coli verotoxin positive, Salmonella spp., Shigella spp. + enteroinvasive Escherichia coli, Yersinia enterocolitica, Clostridium difficile*. Two different positives controls for viral and bacterial multiplex RT-qPCR reactions and a negative control tube are provided in these kits.

### Statistical analysis

Quantitative variables were described by using means [Min-Max] and standard deviations were compared by the Wilcoxon test. Qualitative variables were described by using proportions and compared using a chi-square or Fisher’s exact test if the chi-square test was not applicable; the results were presented as odds ratio with 95% confidence intervals (OR [95% CI]). We used unconditional logistic regression model to study the factors associated with SGI in ARI patients (yes/no) by comparing independent effects of factors that were associated in the bivariate analyses (*p*-value of <0.20). Variables for the model were chosen through automatic backwards selection using a significance level of 0.05. Bivariate and multivariate analyses were performed on patients with only one pathogen detected in nasopharyngeal swabs and/or in stool sample. All analyses were been performed using the R program (http://www.r-project.org).

## Results

**Fig. 1:**
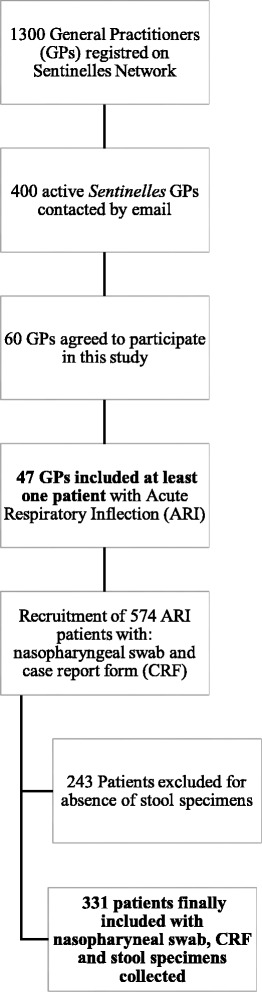
Flow diagram describing selection of patients included in the study

During the study period, 47 of the 60 recruited GPs (78.3%) that agreed to participate in the study enrolled at least one ARI patient. Of the 574 ARI patients recruited by these GPs, 331 (57.6%) sent a stool sample to the virology laboratory and finalized their inclusion in this study (Fig. 1). There were no significant differences in socio-demographic and clinical characteristics or positivity rate for the analysed pathogens between the 574 ARI patients initially recruited by the GPs and the 331 ARI patients with nasopharyngeal and stool samples and completed CRF who were finally included (Table 1).

**Table 1:** Comparison of demographical, clinical and microbiological characteristics between patients initially recruited by General Practitioners (GPs) with at least a nasopharyngeal swab (*N* = 574) and patients included in the study as presenting a nasopharyngeal swab and a stool specimen (*N* = 331)

Characteristics	Patients with at least a nasopharyngeal sample N (%)	Patients with nasopharyngeal and stool sample N (%)	*P*-value*
Number of samples	574	331	
Male	268 (46.7)	154 (46.5)	*P* > 0.05
Mean age [Min-Max]	*35.9 [1-91]*	*35.8 [1 - 91]*	*P* > 0.05
0-4 y	38 (6.6)	25 (7.5)	*P* > 0.05
5-14 y	66 (11.5)	59 (17.8)	*P* > 0.05
15-44 y	241 (42)	122 (36.8)	*P* > 0.05
45-64 y	141 (24.6)	86 (26)	*P* > 0.05
≥ 65 y	66 (11.5)	39 (11.8)	*P* > 0.05
Vaccination against influenza	51 (8.9)	36 (10.9)	*P* > 0.05
Travel (<15 days)	29 (5)	20 (6)	*P* > 0.05
Risk factor	223 (38.8)	124 (37.5)	*P* > 0.05
Chronic disease	189 (32.9)	106 (32)	*P* > 0.05
Depression	51 (8.9)	26 (7.8)	*P* > 0.05
Hospitalization	35 (6.1)	20 (6)	*P* > 0.05
Digestive disorders <7 days	103 (17.9)	59 (17.8)	*P* > 0.05
*Symptoms*			
High Fever (>39 °C)	275 (47.9)	156 (47.1)	*P* > 0.05
Asthenia	507 (88.3)	288 (87)	*P* > 0.05
Myalgia	431 (75.1)	243 (73.1)	*P* > 0.05
Headaches	424 (73.9)	244 (73.1)	*P* > 0.05
Otitis	62 (10.8)	28 (8.5)	*P* > 0.05
Dyspnea	127 (22.1)	73 (22)	*P* > 0.05
Cough	510 (88.8)	299 (90.3)	*P* > 0.05
Expectoration	196 (31.2)	103 (31.1)	*P* > 0.05
Rhinitis	426 (74.2)	251 (75.8)	*P* > 0.05
Pharyngitis	338 (58.9)	198 (59.8)	*P* > 0.05
Hyperemia	150 (26.1)	81 (24.5)	*P* > 0.05
Adenopathy	66 (11.5)	35 (10.6)	*P* > 0.05
Dehydration	6 (1)	4 (1.2)	*P* > 0.05
Gastrointestinal symptoms (SGI)	327 (57)	189 (57.1)	*P* > 0.05
Diarrhea	84 (14.6)	47 (14)	*P* > 0.05
Vomiting	66 (11.5)	28 (8.5)	*P* > 0.05
Nausea	195 (34)	105 (31.7)	*P* > 0.05
Abdominal pain	197 (34.3)	113 (34.1)	*P* > 0.05
*Food consumption*			
Raw shellfish and molluscs	37 (6.4)	25 (7.5)	*P* > 0.05
Cooked shellfish and molluscs	51 (8.9)	31 (9.4)	*P* > 0.05
Tap water	351 (61.5)	198 (59.8)	*P* > 0.05
*Drug consumption before consultation*			
Antibiotics	29 (5)	17 (5.1)	*P* > 0.05
Antiviral	16 (2.8)	8 (2.4)	*P* > 0.05
Anti-inflammatory	86 (14.9)	46 (13.9)	*P* > 0.05
Antipyretics	331 (57.7)	189 (57.1)	*P* > 0.05
Other drugs	104 (18.1)	64 (19.3)	*P* > 0.05
*Drug prescription after consultation*			
Antibiotics	104 (18.1)	57 (17.2)	*P* > 0.05
Antiviral	48 (8.4)	24 (7.2)	*P* > 0.05
Antipyretics	473 (82.4)	271 (81.9)	*P* > 0.05
Other drugs	165 (28.7)	103 (31.1)	*P* > 0.05
*Results of virological analyses in nasopharyngeal samples*			
Nasopharyngeal samples positive to at least one pathogen	320 (55.7)	204 (61.6)	*P* > 0.05
Influenza (A + B)	176 (30.7)	114 (34.4)	*P* > 0.05
Influenza A (including 4 A not subtyped)	69 (12)	42 (12.7)	*P* > 0.05
A(H1N1)pdm09	36 (6.3)	24 (7.2)	*P* > 0.05
A(H3N2)	29 (5)	14 (4.2)	*P* > 0.05
Influenza B	107 (18.6)	72 (21.7)	*P* > 0.05
Influenza B Victoria	89 (15.5)	57 (17.2)	*P* > 0.05
Influenza B Yamagata	18 (3.1)	15 (4.5)	*P* > 0.05
Human *Coronaviruses* NL63, 229E, OC43, HKU1	48 (8.4)	35 (10.6)	*P* > 0.05
Human Rhinovirus	49 (8.5)	25 (7.5)	*P* > 0.05
Respiratory Syncytial virus A/B	26 (4.5)	20 (6)	*P* > 0.05
Human *Bocavirus*	5 (0.9)	3 (0.9)	*P* > 0.05
Human Metapneumovirus A/B	16 (2.8)	9 (2.7)	*P* > 0.05
Human Parainfluenza viruses 1, 2,3,4	6 (1)	4 (1.2)	*P* > 0.05
Human Adenovirus	8 (1.4)	4 (1.21)	*P* > 0.05
Human *Parechovirus*	0	0	
Mycoplasma pneumoniae	0	0	
Enteroviruses	0	0	
Co-infection	13 (2.3)	10 (3)	*P* > 0.05

*P-value resulted of Chi square or Fisher exact test

The demographic data and clinical characteristics of the 331 ARI cases studied are summarized in Table 1. At least one GI symptom was declared by 189 (57.1%) of the 331 ARI patients: diarrhea was reported by 47 (14%), vomiting by 28 (8.5%), nausea by 105 (31.7%) and abdominal pain by 113 (34.1%) (Table 1).

### Respiratory pathogens identified in nasopharyngeal samples

**Fig. 2:**
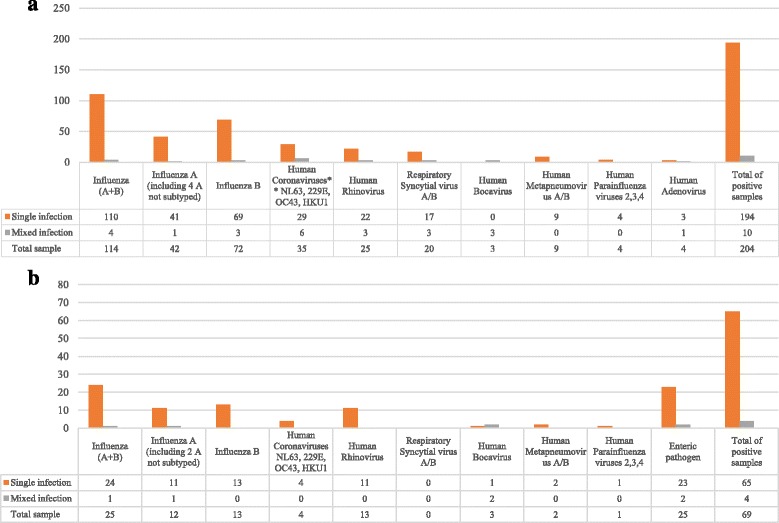
**a** Description of number of positive nasopharyngeal specimens to at least one respiratory pathogen and **b)** description of number of positive stool specimens to at least one respiratory or enteric pathogen. **a** * Single infection rate for nasopharyngeal samples was of 87.25% (194/204) and multiple infection rate was of 5% (10/204). ** HCoV details: among 35 positive samples we detected: 13 NL63, 10 229E, 7 OC43 and 5 HKU1. **b** * Among 25 patients with positive stools to influenza A or B viruses, one patient with influenza B had negative nasopharyngeal sample. **Single infection rate for stool samples was of 94.2% (65/69) and multiple infection rate was of 5.8% (4/69). *** HCoV details: among 4 positive samples we detected: 1 OC43 and 2 NL63 and 1 229E

Overall, the nasopharyngeal specimens of 204 of the 331 (61.6%) patients were positive for at least one of the 12 respiratory pathogen groups analysed in this study (Table 1). Infection with a single virus accounted for 87.2% (194/204) of the positive nasopharyngeal samples, whereas infections with multiple viruses observed in 5% (10/204) of them, including nine double infections: (A(H1N1)pdm09/HCoV, ADV/HBoV, two Influenza B virus/HCoV, two HCoV/HRVS and two HRV/HBoV) and one triple infection (HRV/ADV/HRSV) (Fig. 2a). The most frequently identified pathogen was influenza virus (34.4%, 114/331; consisting of influenza A virus [12.7%, 42/331] and influenza B virus [21.7%, 72/331]), followed by HCoV (10.6%, 35/331), HRV (7.5%, 25/331) and RSV (6.0%, 20/331) (Table 1 and Fig. 2a). Of the 35 samples that tested positives for HCoV, 13 were HCoV-NL63, 10 HCoV-229E, 7 HCoV-OC43 and 5 HCoV-HKU1.

### Respiratory and enteric pathogens identified in stool samples

Of the 331 stool samples, 69 (20.8%) were positive for at least one pathogen (respiratory and/or enteric) (Fig. 2b). Of the 69 positive stool samples, 94.2% (65/69) were positive for a single pathogen, whereas multiple viruses (all double infections) were detected in 5.8% (4/69) of positive stool specimens (A(H3N2)/ADV, HBoV/ADV and two HRV/HBoV) (Fig. 2b).

The percentage of positive patients was highest for influenza viruses (7.5%, 25/331), for enteric pathogens (7.5%, 25/331) followed by HRV (3.9%, 13/331). HCoV (1 HCoV-NL63, 1 HCoV-229E, 1 HCoV-OC43 and 1 HCoV-HKU1), HBoV, HMPV and PIV were detected in fewer than 2% of the 331 stool specimens from ARI cases (Fig. 2b).

#### Factors related to GI symptoms

All factors listed in Table 1 have been analysed to investigate association with GI symptoms in ARI patients. Table 2 shows factors significantly related to GI symptoms in ARI patients. Results of RNA/DNA positivity in stools between ARI patients with and without GI symptoms for the respiratory pathogens tested were also reported in Table 2.

**Table 2:** Odds ratios and 95% confidence intervals bivariate and multivariate models of the risk of gastrointestinal (GI) symptoms among 331 patients with acute respiratory infections (ARIs)

Characteristics	ARI patients With GI symptoms *N* = 189 (%)	ARI patients Without GI symptoms *N* = 142 (%)	OR^a^ [95% IC] (*p*-value)	aOR^b^ [95% CI] (*p*-value)
High Fever (>39 °C)	99 (52.4)	57 (40.1)	1.6 [1.1-2.5] (0.01)	1.7[1.1-2.7] (0.03)
Headaches	151 (79.9)	93 (65.6)	2 [1.2-3.4] (0.002)	2 [1.2-3.4] (0.003)
Human Coronavirus in single infection in nasopharynx	22 (11.6)	6 (4.2)	2.9 [1.1-7.6] (0.01)	2.7 [1.2-6.8] (0.02)
Antipyretic consumption before consultation among 104 influenza patients without co-infection	32/59 (54.2)	35/45 (77.8)	0.3 [0.1-0.8] (0.001)	0.3 [0.1-0.6] (0.002)
***Pathogens detected in stools***				
Influenza A	8 (4.2)	4 (2.8)	1.5 [0.4-5.2] (0.46)	n.i.
Influenza B	6 (3.1)	7 (4.9)	0.6 [0.2-1.9] 0.66	n.i
Human Rhinovirus	6 (3.1)	7 (4.9)	0.6 [0.2-1.9] (0.66)	n.i
Human Coronavirus	3 (1.6)	1 (0.7)	2.3 [0.3-22.1] (0.42)	n.i
Respiratory Syncytial Virus	0 (0.0)	0 (0.0)	n.i.	n.i
Human Bocavirus	1 (0.5)	0 (0.0)	n.i.	n.i
Human Metapneumovirus	1 (0.5)	1 (0.7)	n.i.	n.i
Human Parainfluenzavirus	1 (0.5)	0 (0.0)	n.i.	n.i
All respiratory pathogens	26 (13.7)	20 (14.1)	0.9 [0.5 1.9] (1)	n.i
Enteric pathogens	19 (10.0)	5 (3.5)	3.0 [1.2-8.4] (0.02)	3.2 [1.2-9.9] (0.02)

^a^Crude odds ratios (OR) from bivariate models

^b^aOR = Adjusted odds ratios from multivariate models

*CI* confidence interval, *n.i.* =not included in the model

ARI patients who reported at least one GI symptom (57.1%; 189/331) were associated with the presence of high fever (>39 °C) (adjusted odds ratio [aOR] = 1.7 95% confidence interval [CI] [1.1–2.7]; *p* = 0.03), and headaches (aOR = 2.0 [1.2–3.4]; *p* = 0.003) (Table 2).

ARI patients with GI symptoms were more likely to have at least one enteric infection (aOR = 3.2 [1.2–9.9]; *p* = 0.02) detected in stool or to have an infection with HCoV detected in the nasopharynx (aOR = 2.7; [1.2–6.8]; *p* = 0.002) (Table 2). Proportion of GI symptoms in ARI patients with single infection ranged from 33.3% with HRV infection (in nasopharyngeal swab) to 79.2% with enteric pathogens infection (in stool specimens) (Table 3). ARI patients with HCoV detected in the nasopharynx or enteric pathogen detected in stool were statistically more likely to have GI symptoms than ARI patients with other respiratory pathogens infection (Table 3). Among the 104 ARI patients with laboratory-confirmed influenza at least in the nasopharynx, 56.7% (59/104) had GI symptoms (Table 2). Consumption of antipyretic medication before the consultation seemed to reduce the risk of developing GI symptoms for this population (aOR = 0.3 [0.1–0.6]; *p* = 0.002) (Table 2).

**Table 3:** Proportion of gastrointestinal (GI) symptoms in patients with acute respiratory infections (ARIs) with single virus infection

	Human Coronavirus*	Influenza A*	Influenza B*	Human Rhinovirus*	Respiratory syncytial virus*	Human Adeno virus*	Human Bocavirus*	Human Metapneumovirus*	Human Parainfluenzavirus *	Enteric pathogens **	*p-value*
Number of single virus detection in ARI patients	28	41	63	21	14	3	0	9	4	24	
GI symptoms in ARI patients with single virus detection N (%)	**22 (78.6)**	22 (53.6)	35 (57.1)	*7 (33.3)*	6 (42.8)	2 (66.6)	0	5 (55.5)	3 (75)	**19 (79.2)**	*0.02* ^*a,b*^

*in nasopharyngeal swab

**in stool specimen

Significant differences are noted as **bold** (Highest) versus *italic* (lowest) when possible

^a^Pearson Chi-square test

^b^interpret with caution as any cell as a value < 5

## Discussion

In this study, results showed that the presence of GI symptoms in ARI patients could not be explained by the detection of respiratory pathogens in stools. However, GI symptoms were more common among patients with ARI who were exclusively infected with HCoV detected in nasopharyngeal sample. This association cannot be explained by the presence of HCoVs in stools because the simultaneous detection of HCoV in nasopharyngeal and stool specimens was sporadic.

Even if the association of GI symptoms with enteric infections is not surprising, it is interesting to point out that 13.2% (25/189) of ARI infections with GI symptoms were associated with laboratory-confirmed enteric infections. This result suggests that GI symptoms in patients with ARI could be related to enteric infections, and that the positive correlation between GI symptoms and fever or headache observed in this study increases the difficulty of clinical diagnosis.

We detected, HCoVs in 10.6% of nasopharyngeal samples of patients with ARI. These results are in line with previous studies reporting HCoVs in 2.1%–18% of respiratory samples [27] of ARI patients. In the present study, patients with HCoVs featured 11.6% of ARI patients with GI symptoms. Moreover 78.9% of patients with HCoV infection declared to have GI symptoms. Although HCoVs are recognized as causes of respiratory infection, their role in gastrointestinal infection remains uncertain and a subject of debate [12, 28, 29]. In the present study, GI symptoms were positively associated with single laboratory-confirmed HCoV infection detected in the nasopharynx of ARI patients. This association cannot be explained by the presence of HCoVs in stools because the simultaneous detection of HCoV in nasopharyngeal and stool specimens was observed in four patients only. The four commonly circulating HCoVs (1 HCoV-NL63, 1 HCoV-229E, 1 HCoV-OC43 and 1 HCoV-HKU1) were detected in stool samples, thus none of the four HCoV could be specifically associated with positivity of stools. The proportion of HCoVs in stool specimens was less important than it was in nasopharyngeal specimens (4 versus 28 respectively) which hampered an efficient comparison of the results and limited their interpretation. Moreover there was no ARI patient presenting HCoV in stools in the absence of HCoV in nasopharynx. Therefore the presence of HCoV RNA in stool is likely due to swallowing rather than due to local replication in the GI tract [12]. The presence of HCoVs in nasopharynx seems to be linked to GI symptoms in ARI patients but the biological mechanism remained unclear. In line with previous studies [13], no association was observed between seasonal influenza virus detection in nasopharyngeal or stool samples and GI symptoms in ARI patients. However, among the 104 patients with influenza infection, 56.7% (59/104) presented GI symptoms. The mouse model used by Wang [20] showed that influenza infection through a mechanism dependent on type I interferons (IFN-Is) can alter the composition of the intestinal microbiota, resulting in immunological dysregulation that may promote inflammatory gut disorders. The number of *Escherichia coli (E.coli)* in the intestinal tract increased, perhaps leading to intestinal immune injury. A similar study [30] reported that influenza-induced IFN-Is enhance susceptibility to Salmonella intestinal colonization and dissemination during secondary Salmonella-induced colitis through suppression of host intestinal immunity. The systemic role for IFN-Is in altering the intestinal microbial balance after influenza infection need to be explored.

Interestingly, we found that the consumption of antipyretic drugs before consultation seemed to reduce the risk of developing GI symptoms among laboratory-confirmed influenza patients. This result is in line with previous studies that showed that paracetamol dramatically decreases the morbidity associated with influenza, thereby reducing the clinical symptoms associated with influenza virus infection [31, 32]. Therefore, the consumption of antipyretic drugs before consultation may lead to the underestimation of the frequency of GI symptoms in patients with laboratory-confirmed influenza.

The strengths of this study include its prospective multicentrer design and study length spanning two consecutive ARI seasons, standardized patient screening by the participant GPs, centralized confirmation of microbiological data, the simultaneous search of respiratory pathogens in nasopharyngeal and stool samples and the presence of enteric pathogens (viruses and bacteria) in stool, and other confounding factors that might also cause GI symptoms.

This study did have several limitations. First, the main limitation of this study was the lack of culturing of respiratory viruses from stool samples to determine if RT-qPCR detection represented the presence of viable virus. The detection of respiratory viruses in the stool could simply be RNA/DNA from viruses that were swallowed. A recent study showed that a swallowed virus could be detected in stools if protective mechanisms render it resistant to gastric acid and bile/pancreatic juice [33]. High viscosity of mucus could protect influenza viruses from inactivation in the gastrointestinal environment, accounting for detection of the virus in feces [33]. Second, the number of patients included here did not allow the identification of meaningful associations by sub-analyses. Studies with a small-to-moderate sample size that employ logistic regression have been reported to overestimate the effect measure [34]. Third, we did not collect data pertaining to GI symptoms after GP consultation, which hampered the interpretation of the results.

## Conclusion

In conclusion, except for ARI patients with enteric pathogens in stool samples, the presence of GI symptoms in ARI patients could not be explained by the detection of respiratory pathogens in stools. However, the detection of enteric pathogens in stool samples could explained by the presence of GI symptoms in some of ARI cases. The biological mechanisms explaining the association between the presence of HCoVs in nasopharynx and GI symptoms need to be explored.

## Additional files

Additional file 1: Gastrointestinal symptoms’ proportion by respiratory pathogens infection found in previous studies. (PDF 210 kb)

Additional file 2: Detection number and/or detection rate of respiratory viruses in stool of Acute Respiratory Infection (ARI) patients found in previous studies. (PDF 93 kb)

Additional file 3: **a)** Seasonal distribution of influenza viruses identified in patients consulting for an Acute Respiratory Infection (ARI) during 2014-2015 season and 2015-2016 season. (PDF 389 kb)

Additional file 4: List of information collected by General Practitioners in the Case Report Form during consultation recruiting of patients with ARI. (DOCX 20 kb)

## Abbreviations

ARI: Acute Respiratory Infection
CRF: Case report form
EV: Enterovirus
FDT: Fast track diagnostic
GI: Gastrointestinal
GP: General practitioner
HAdV: Human adenovirus
HBoV: Human Bocavirus
HCoV: Human Coronavirus
HMTP: Human Metapneumovirus
HPeV: Human Parechovirus
HPIV: Human Parainfluenza virus
HRV: Human Rhinovirus
M.pneu: *Mycoplasma pneumoniae*
RSV: Respiratory Syncytial virus
RT-qPCR: Real-time Reverse Transcription-polymerase Chain Reaction test
