# Supplementary material for: Clinical and virological factors associated with gastrointestinal symptoms in patients with acute respiratory infection: a two-year prospective study in general practice medicine
Source: BMC Infect Dis. 2017 Nov 22;17:729. doi: 10.1186/s12879-017-2823-9 (PMC5700681; doi:10.1186/s12879-017-2823-9)
Supplement: Supplementary file 1 — Gastrointestinal symptoms’ proportion by respiratory pathogens infection found in previous studies. (PDF 210 kb) [file 12879_2017_2823_MOESM1_ESM.pdf]

Additional file 1 : Gastrointestinal symptoms' proportion by respiratory pathogens infection found in previous studies.

Pathogens	Population	Symptoms			
Human Adenovirus		SGI (no details)	Diarrhea	Vomiting	Abdominal pain
(1)	Children with acute respiratory infections		50% (3/6)		
(2)	All ages population hospitalized with acute lower respiratory infection		5.6% (54/957)		1.4% (13/957)
Human Bocavirus					
(4)	Hospitalized children with respiratory tract infection	10.6% (7/66)			
(2)	All ages population hospitalized with acute lower respiratory infection		11.6% (64/551)		1.3% (7/551)
Human Coronavirus					
(4)	Hospitalized children with viral respiratory infection		10.9% (7/64)	26.6% (17/64)	
(2)	All ages population hospitalized with acute lower respiratory infection		4.6% (18/393)		1% (4/393)
Human Influenza virus					
(2)	All ages population hospitalized with acute lower respiratory infection		5.1% (96/1869)		1.6% (30/1869)
(5)	Children with acute respiratory infections	60% (12/20)			
Influenza A virus					
(8)	Hospitalized children with Influenza A infection		26% (11/42)	52% (22/42)	
(1)	Children with acute respiratory infections		3.7% (n=27)		
Seasonal Influenza B virus					
(6)	Hospitalized children with Influenza B infection		14% (2/14)	36% (5/15)	
Human Metapneumovirus					
(7)	Hospitalized children with acute respiratory tract infection	50%			
(3)	Hospitalized children with respiratory tract infection	8.2% (4/49)			
Human Parainfluenza virus					
(1)	Children with acute respiratory infections		0%		
(3)	All ages population hospitalized with acute lower respiratory infection		11.1% (151/1366)		1.5% (20/1366)
Human Rhinovirus					
(8)	Residents of a long-term care facilities	34% (n=33)			
(3)	Hospitalized children with respiratory tract infection	5.7% (12/211)			
VRS					
(3)	Hospitalized children with respiratory tract infection	9.8% (14/143)			
(6)	Hospitalized children with VRS		12% (14/119)	38% (45/119)	
