# Supplementary material for: Clinical and virological factors associated with gastrointestinal symptoms in patients with acute respiratory infection: a two-year prospective study in general practice medicine
Source: BMC Infect Dis. 2017 Nov 22;17:729. doi: 10.1186/s12879-017-2823-9 (PMC5700681; doi:10.1186/s12879-017-2823-9)
Supplement: Supplementary file 2 — Detection number and/or detection rate of respiratory viruses in stool of Acute Respiratory Infection (ARI) patients found in previous studies. (PDF 93 kb) [file 12879_2017_2823_MOESM2_ESM.pdf]

Additional file 2 : Detection number and/or detection rate of respiratory viruses in stool of Acute Respiratory Infection (ARI) patients found in previous studies.

Pathogens	Population	Number or % Stool positive
**Human Bocavirus**		
(11)	Hospitalized children with acute respiratory tract infection	52.9 % of all 34 positive cases
**Human Coronavirus**		
(12)	Hospitalized children with acute gastroenteritis or respiratory tract infection	2 % (19/955)
**Human Influenza virus**		
(5)	Hospitalized children with influenza infection	2 patients
(13)	Adults hospitalized with laboratory confirmed influenza A infections	Influenza A viral RNA was detected in 56 of 119 (47%) of stool samples, 59% were A(H1N1) and 49% were A(H3N2)
**Human Metapneumovirus**		
(14)	Hospitalized children	0%
**Human Rhinovirus**		
(10)	Children with upper respiratory infection	35.1% (149/425)
**VRS**		
(14)	Hospitalized children	13% (5/37)
