# Supplementary material for: Clinical and virological factors associated with gastrointestinal symptoms in patients with acute respiratory infection: a two-year prospective study in general practice medicine
Source: BMC Infect Dis. 2017 Nov 22;17:729. doi: 10.1186/s12879-017-2823-9 (PMC5700681; doi:10.1186/s12879-017-2823-9)

Additional file 3: **a)** Seasonal distribution of influenza viruses identified in patients consulting for an Acute Respiratory Infection (ARI) during 2014-2015 season and 2015-2016 season.

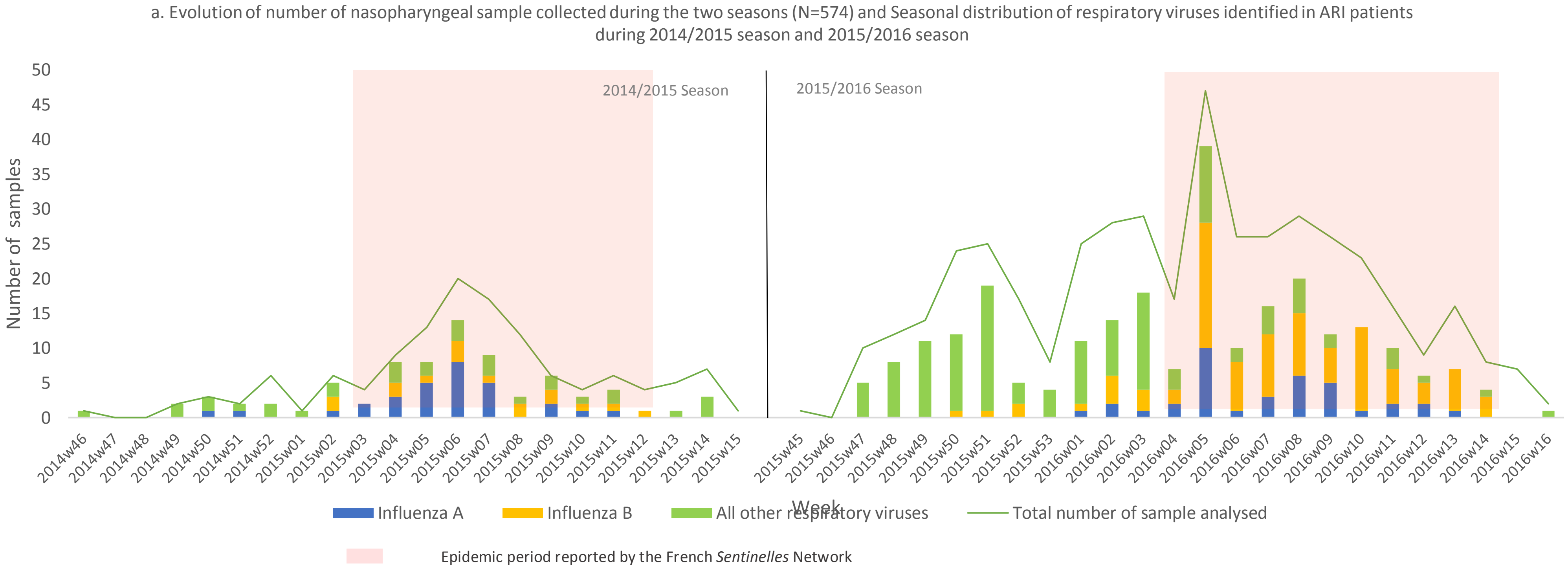

Supplement: Supplementary file 3 — a) Seasonal distribution of influenza viruses identified in patients consulting for an Acute Respiratory Infection (ARI) during 2014-2015 season and 2015-2016 season. (PDF 389 kb) [file 12879_2017_2823_MOESM3_ESM.pdf]
