# Supplementary material for: Clinical and virological factors associated with gastrointestinal symptoms in patients with acute respiratory infection: a two-year prospective study in general practice medicine
Source: BMC Infect Dis. 2017 Nov 22;17:729. doi: 10.1186/s12879-017-2823-9 (PMC5700681; doi:10.1186/s12879-017-2823-9)
Supplement: Supplementary file 4 — List of information collected by General Practitioners in the Case Report Form during consultation recruiting of patients with ARI. (DOCX 20 kb) [file 12879_2017_2823_MOESM4_ESM.docx]

**Additional file 4**: List of information collected by General Practitioners in the Case Report Form during consultation recruiting of patients with ARI.

| 1. Consultation’s date |
| --- |
| 1. Age and sex of patient |
| 1. Professional status |
| 1. Vaccination status against Influenza for the current year |
| 1. Digestive disorders less than 7 days before consultation |
| 1. Travel less than 15 days before consultation |
| 1. Consumption of cooked or raw oysters, mussels or shellfish |
| 1. Consumption of tap water |
| 1. Consumption of drug 7 days before consultation:  - Antiviral - Antibiotic - Anti-inflammatory - Antipyretics - Other |
| 1. Influenza Like-Illness signs and symptoms:  - High Fever (>39°C) - Asthenia - Myalgia - Headaches - Otitis - Dyspnea - Cough - Expectoration - Rhinitis - Pharyngitis - Hyperaemia - Adenopathy - Dehydration |
| 1. Gastrointestinal symptoms:  - Diarrhea - Vomiting - Nausea - Abdominal pain |
| 1. Chronic disease |
| 1. Risk factor for severe influenza:  - Obesity - Health worker - Infant - Pregnancy |
| 1. Depression |
| 1. Hospitalisation |
| 1. Medical prescription:  - Antiviral - Antibiotic - Antipyretic - Other |
